# Peer mentor versus teacher delivery of a physical activity program on the effects of BMI and daily activity: protocol of a school-based group randomized controlled trial in Appalachia

**DOI:** 10.1186/s12889-018-5537-z

**Published:** 2018-05-16

**Authors:** Laureen H. Smith, Rick L. Petosa, Abigail Shoben

**Affiliations:** 1 The Ohio State University College of Nursing, 240 Newton Hall, 1595 Neil Avenue, Columbus, OH 43201 USA; 2 The Ohio State University College of Education and Human Ecology, Physical Activity and Exercise Science Room 048, Columbus, OH 43201 USA; 3 The Ohio State University College of Public Health, 249 Cunz Hall, Columbus, OH 43201 USA

**Keywords:** Peer mentoring, Physical activity, BMI, Appalachia

## Abstract

**Background:**

Rural Appalachian populations have poorer health and fewer positive health-related behaviors compared to other United States populations. Appalachians are the most sedentary U.S. population and teens are particularly sedentary. Obesity prevention through improving physical activity is a top priority in Rural Healthy People 2020. Obesity prevalence among Appalachian teens exceeds the national rates of 13.9% and has consistently been greater than 26%. Organized sports has not been effective at improving daily physical activity or health outcomes for Appalachian teens. The purpose of this study is to test the efficacy of a 10-week school-based intervention in promoting self-regulation of physical activity among adolescents not participating in organized sports. By using accelerometers, our study will measure both sedentary time and planned exercise during waking hours.

**Methods:**

The design for this four-year study is a **group-randomized controlled trial** (G-RCT). We will recruit high schools in 3 waves, with 4 in Wave 1, 8 in Wave 2, and 8 in Wave 3, for a total of 20 schools. For each wave of schools, we will randomly assign half of the schools to each condition--intervention (peer-to-peer mentoring [MBA]) and comparison (teacher-led [PBA])--for a total of 10 schools in each of the two conditions by study’s end. We will collect data at baseline (T_1_), 3 months post intervention (T_2_), and 6 months post intervention (T_3_). Linear Mixed Models (LMMs) and Generalized Linear Mixed Models (GLMMs) will be used to test the main hypotheses. Power for this study was based the primary analysis comparing BMI outcomes at T_2_ between the groups, adjusting for baseline BMI values.

**Discussion:**

This study provides age-appropriate lifestyle education and skill building. Peer-to-peer mentoring by local high school students and school-based tailored support strengthens sustainable behavioral change. Focusing on unique healthy-lifestyle challenges prevalent in low-resource areas such as Appalachia such as overcoming environmental, social, and psychological barriers may improve adherence to physical activity. Serving as role models, peer mentors may improve their own lifestyle behaviors, providing a dual intervention.

**Trial registration:**

NCT02329262.

## Background

Rural Appalachian populations have poorer health and fewer positive health-related behaviors [1–4] compared to other United States populations [2, 5, 6]. Appalachians are the most sedentary U.S. population [7–9], and teens are particularly sedentary. Nationally 27% of adolescents reported 60 min of daily physical activity, while less than 20% of U.S. adolescents report engaging in 60 min or more of daily vigorous physical activity [10]. Residents of Appalachia face disproportionate burdens to engage in daily physical activity [5]. A persistent pattern of disproportionate low daily physical activity begins in youth and intensifies into adulthood [5].

Obesity prevention through improving physical activity is a top priority in Rural Healthy People 2020 [11]. Obesity prevalence among Appalachian teens exceeds the national rates of 13.9% and has persistently been greater than 26% [10–16]. The high prevalence of obesity combined with high rates of sedentary behaviors place Appalachian teens at increased risk for development of poor health outcomes later in life. Compared to other Americans, Appalachians are less likely to be physically active in their leisure time [17, 18]. Similarly, though most teens in the United States receive less physical activity than is recommended, in rural, under-resourced areas of Appalachia, sedentary activity rates are significantly higher than national levels [2, 10, 17–19].

Statistically, the Appalachian region lags behind the nation in most socioeconomic and health indicators, including: poverty, housing, education, employment, access to care, and quality of life [4, 8, 20–22]. In academic underperforming schools, such as those prevalent in Appalachia, the primary educational focus is to meet core academic mandates. To meet academic mandates, most Appalachian schools no longer require health and physical education for graduation. Efforts to improve physical activity in school-aged Appalachian adolescents have relied on organized sports. School-sponsored sports programs only engage a small percentage of high school students. Relying on organized sports has not been effective at improving daily physical activity or health outcomes for Appalachian teens [22, 23]. One explanation may be unique circumstances present in Appalachia. Opportunities to participate in organized sports are limited due to inadequate school resources, lack of transportation, and limited availability of school teams [23]. As a result, most adolescents residing in Appalachia are unable to participate in organized sports.

Further, school-based health interventions are limited in their scope and impact on obesity prevention [1, 3, 24–27]. School-based interventions typically deliver content as part of a regular course such as health or physical education via teachers in classroom settings. Low efficacy of these programs may be due to unique cultural challenges [28] such as a preference for informal sharing of information among local residents rather than health content delivered by formal teachers [6, 21]. Though school-based interventions increase health knowledge, there is less evidence of the effectiveness for health behavior changes leading to obesity prevention [1, 21, 24–27]. Longer-term follow-up of health behavior and health status outcomes in intervention studies also are lacking [1, 21, 26].

Our NIH-funded study (R01080866) expands school-based intervention research by testing the efficacy of a 10-week school-based intervention in promoting self-regulation of physical activity among adolescents. By using accelerometers, our study will measure both sedentary time and all forms of physical activity during waking hours. This study extends the science by following adolescents during the summer months post intervention, thus capturing self-regulation of behavioral changes and health outcomes (baseline, 3-month follow-up, and 6-month follow-up). By using local residents as peer mentors and teachers for intervention delivery, our study’s impact extends to the surrounding community and provides a foundation for long-term sustainability of the program.

This study will use the *Planning to be Active* (PBA) curriculum, a physical activity program designed for delivery in a classroom setting. For this study, the curriculum is adapted to also be delivered via trained peer mentors over a 10-week period for 40 min each week per session. The adapted version is called *Mentored Planning to be Active* (MBA)*.* Adaptations for MBA include: (a) extending the curricular time to 40 min; (b) incorporating mentor-led activities via Discussion Guides; and (c) engaging in individual and group physical activity.

### Peer mentoring delivery approach

Our study is designed to overcome challenges unique to Appalachia [1, 3] by using trained teen residents to provide health education, skills, and support to enable health behavior change. Mentoring approaches have been effective at addressing health-risk behaviors among Appalachian youth [29, 30], including overweight and obesity [14, 31]. In rural Appalachia, cross-age mentors (older children mentoring younger) helped mentees improve academic achievement and positive connectedness to parents and family [32]. While serving as academic mentors to children, teens reported improvements on their own academic and self-esteem outcomes [30, 33]. Smith and Holloman found that Appalachian peer mentors helped mentees improve nutritional knowledge and dietary behaviors on a short-term basis [14, 31]. Appalachian youth assigned to peer mentors (versus adult leaders) demonstrated improved BMI and increased physical activity on a short-term basis [31].

The mentoring approach used in our study addresses the Appalachian preference for a less formal social network approach to promoting health behaviors. Our study: (a) expands on the mentoring literature, particularly peer mentoring; (b) extends the use of mentoring for health-promoting and self-regulation behaviors; (c) allows for longer-term follow-up of intervention effectiveness; (d) examines the intervention dose effect; (e) examines the impact of the mentoring experience on behavioral outcomes of the peer mentors; and (f) tests moderator variables to explain the process through which the intervention works best. A group randomized controlled trial (G-RCT) will test the effects on adolescents’ health behaviors and health outcomes of *Planning to Be Active* (PBA) delivered via classroom teachers versus *Planning to Be Active* with peer mentoring delivery (MBA). To better understand the outcomes of the intervention, we will monitor behavioral and health outcomes at the end of the summer months, as well as the dose effect (e.g., number of sessions attended).

Rationale for the peer mentoring delivery approach includes: (a) the recognition that teens spend less time with family members and more time with peers, and (b) the powerful influence that peers have on role modeling and supporting behaviors [33, 34]. In recognition of the importance of family-based support [35, 36], certain PBA and MBA lessons require family-based activity. Further, weekly reinforcement materials include activities to be completed with parents or family members. These activities include home-based recreation and exercise.

The peer mentoring approach used in this study challenges current practice, which relies on adult mentors serving adolescents; adult teachers delivering health curriculum in a classroom setting; and family-based approaches targeting primarily parents of clinically obese children.

Mentors help adolescents overcome personal and social barriers, expose them to new relationships and opportunities, and assist in developing decision making or problem solving skills that facilitate success in everyday life [37, 38]. Mentoring relationships have positively influenced behavior change and health outcomes while promoting positive connections to parents and family, including physical activity [14, 31], academic achievement [30, 39], and substance use/abuse among Appalachian children [33]. Mentoring of teens has resulted in long-term and sustainable behavior change, including reduced substance use [38] and smoking [40]. Mentoring to address other health risks and peer-to-peer mentoring of adolescents is understudied [41].

The use of peer-to-peer mentors to deliver a behavioral self-regulation curriculum to adolescents is an innovative approach to overcoming the unique challenges of this rural, underserved, and economically distressed population. The use of mentors improves existing interventions targeting obesity prevention in Appalachia by providing to adolescents not only information and knowledge, but also tailored support, more personal support, and less formal delivery of health curriculum. Peer mentoring allows for the incorporation of skill-building activities; reinforcement of self-regulating activities; engagement in individual and group physical activity; and support of set weekly goals. Adolescents tend to view peers as: more credible, having a better understanding of the concerns of young people, and being more likely to model the behaviors of peers than adults [30, 32, 36, 41]. Peer mentoring empowers teens by strengthening their social network and social support to plan, regulate, and evaluate their personal activity plan, thus building self-efficacy to engage in regular activity. With peer mentors, physical activity becomes more personalized and tailored to personal interests, talents, and neighborhood environment. These inherent benefits of peer mentoring coupled with the low efficacy of classroom-based health programs with content delivered by teachers led to the development of the MBA approach.

### Theoretical framework of the curriculum

**Fig. 1:**
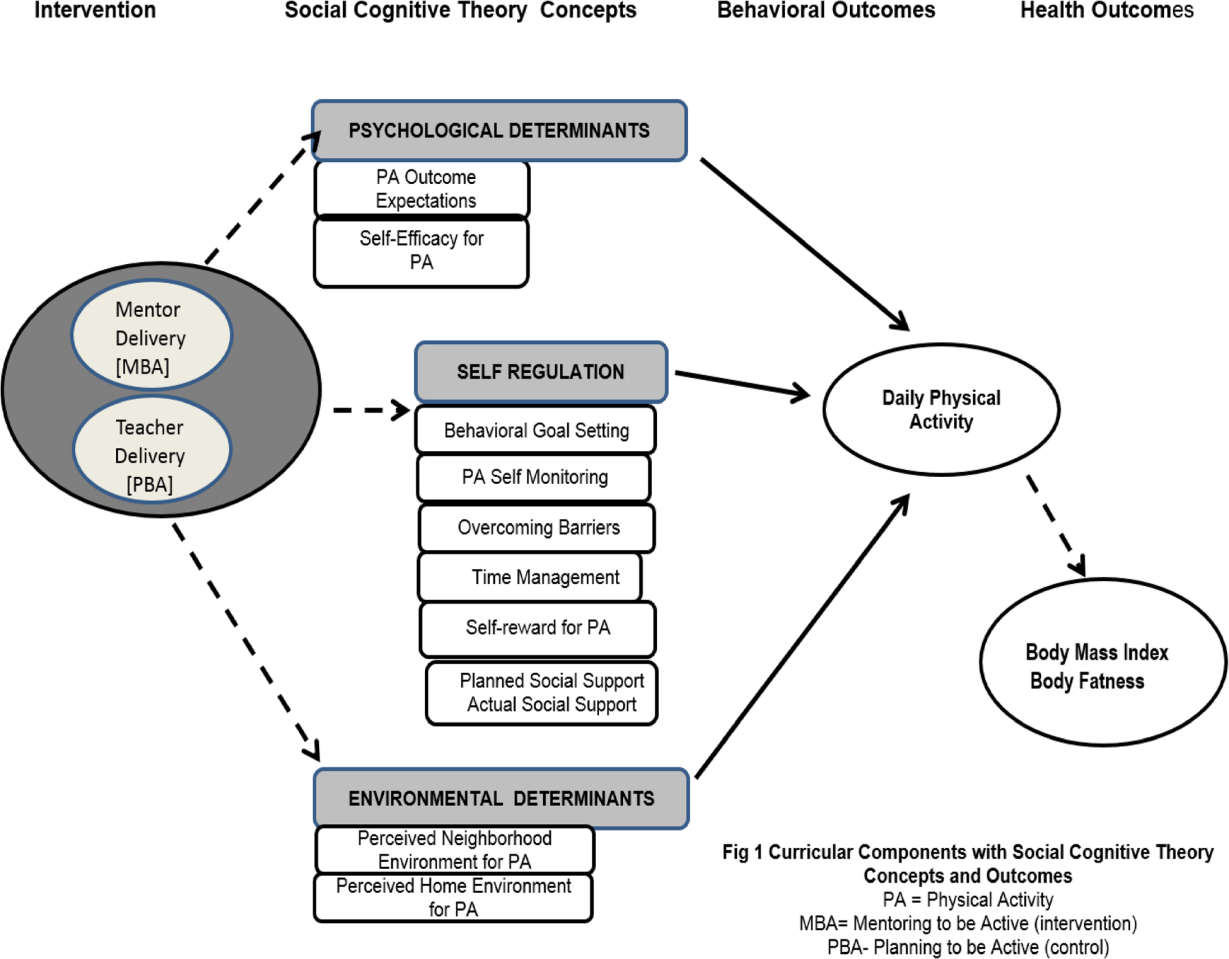
Curricular Components with SCT Concepts and Outcomes

The theoretical framework supporting the curriculum is Social Cognitive Theory (SCT); PBA (comparison curriculum) was developed and tested over the course of 4 intervention studies. These 4 studies revealed: (a) SCT variables were strongly related to moderate and vigorous physical activity; (b) PBA increased self-regulation of physical activity; and (c) a 10-lesson dose led to the greatest improvement in physical activity outcomes. Further, SCT has been used extensively for determinants of physical activity [42] and was used to guide the development of PBA [17–19]. The PBA curriculum is designed to address psychosocial determinants, self-regulation, and environmental determinants affecting individual behavior change (Fig. 1).

Psychosocial determinants include: outcome expectancies and exercise self-efficacy. Self-regulation includes: goal setting, self-monitoring, overcoming barriers, time management, self-reward, and social support. Environmental determinants include: home, neighborhood, and school environmental opportunities for physical activity. The intervention instructs students to actively seek and create supportive environments.

Our experience of working in rural areas of Appalachia suggests that many students will not have access to traditional exercise and fitness facilities characteristic of urban and suburban settings. Lack of transportation and distance to facilities are barriers for many of these students. Therefore, the intervention emphasizes forms of activity that can readily be done in rural neighborhood and home environments. These skills are useful for sustaining goal-directed behavior change. Homework assignments in the PBA curriculum consistently focus on the application and refinement of self-regulation skills applied to the initiation and maintenance of physical activity.

The peer mentoring delivery approach builds and strengthens social networks. Social networks are links between people that provide social support [43, 44]. Social networking provides emotional, informational, and appraisal support that creates a sense of psychological safety (between mentor and mentee), resulting in higher motivation to change behavior. Learning, domain-specific self-efficacy and behavior change are facilitated when people have a sense of psychological safety or the perception that attempts to change behavior can occur without fear or embarrassment [44]. For adolescents in this study, advantages include enhanced learning and behavioral change support resulting from the perceived social support (emotional, informational, and appraisal) and psychological safety promoted by peer-to-peer mentoring.

Because an Appalachian preference is for informal sharing of information among local residents rather than formal health-related behavior change [6, 23, 28] content delivered in a structured classroom, perhaps there is also a preference for receiving such information from those closer in age. Figure 1 depicts the theoretical concepts of SCT hypothesized to affect the outcomes in our study. We predict that by providing intense social support to teens via peer mentoring, curriculum delivery will enhance behavioral outcomes (daily physical activity) and ultimately better health outcomes (BMI, body fatness) compared to a teacher delivering the curriculum in a classroom setting (usual care). We also predict that, by serving as role models, peer mentors will improve their own lifestyle behaviors, providing a duel intervention [39, 45]. This hypothesis is consistent with research suggesting that school-based peer mentoring is effective in changing risk behaviors among children in Appalachia [14, 31, 45].

## Methods

The design for this four-year study is a group-randomized controlled trial (G-RCT). In our situation, students attending the same school are expected to socialize together; thus, a G-RCT is necessary to avoid the risk of cross-contamination. We will recruit high schools in 3 Waves, with 4 in Wave 1, 8 in Wave 2, and 8 in Wave 3, for a total of 20 schools. For each wave of schools, we will randomly assign half of the schools to each condition--intervention (mentoring [MBA]) and comparison (teacher-led [PBA])--for a total of 10 schools in each of the two conditions by study’s end. We will collect data at baseline (T_1_), post intervention (T_2_), and 6 months post intervention (T_3_). Participating schools can be found at Clinical Trials.gov (NCT02329262).

At each school assigned to the PBA delivery, one or two classroom offerings will be held. Each teacher will meet with about 15–25 assigned children in a traditional classroom setting. At each school assigned to MBA delivery, one or two mentoring offerings will be held (e.g., Tuesdays and/or Thursdays). A mentor meets with 2 mentees at each offering; thus, some mentors may see 4 mentees in one week. The Project Directors (PDs) will perform supervisory checks on all sessions; MPIs will perform monthly fidelity checks on sessions. The comparison and intervention groups differ on two factors: teacher/mentor and classroom/mentoring.

Although the individual contributions of these factors cannot be separated, this study allows us to compare the effects of the novel delivery approach (peer mentors) with the usual format (teacher in classroom) and determine impact on outcome measures. To control for seasonal effects, the curriculum will be delivered during the same months (January–March) for all years. Because learning and retention rates decrease during summer, especially for adolescents in low-income families [46–48], reinforcement of critical curricular components will be delivered via one booster session for adolescents via a take-home kit and an interactive website for teen participants in both groups (MBA and PBA) at the end of the academic year (after T_2_ data collection).

### Setting, sample and power analysis

We powered our study based on analysis of the primary outcome: BMI in adolescents at T_3_ (6 months post intervention). We will use a mixed model ANCOVA in our primary analysis. Power in a G-RCT is influenced by five factors: number of groups (schools), number of individuals in each group (adolescents in each school), similarity of outcomes within clusters (school-level intra-class correlation [ICC] for BMI), similarity of outcomes within individuals (correlation of BMI measurements on the same student over time), and percentage of the variance that can be explained by the regression model.

Using data from the **Ohio Family Health Survey**, we estimated that the school-level ICC for BMI among 9th graders in Appalachian Ohio counties is 0.023. We further estimate the over-time correlation of BMI measurements is 0.70 and that adjusting for age and gender will explain approximately 30% of the variance in BMI. With these assumptions, 10 schools per condition, and 50 students per school, we will have 82% power to detect a modest intervention effect (0.2 standard deviation difference between groups). This effect size would correspond to a difference in mean BMI between groups of 1.04 kg/m^2^ if the observed variation in BMI is similar to that of all Appalachian 9th graders from the Ohio Family Health Survey (mean BMI = 23.41, *SD* = 5.2).

### Participant recruitment and retention

**Fig. 2:**
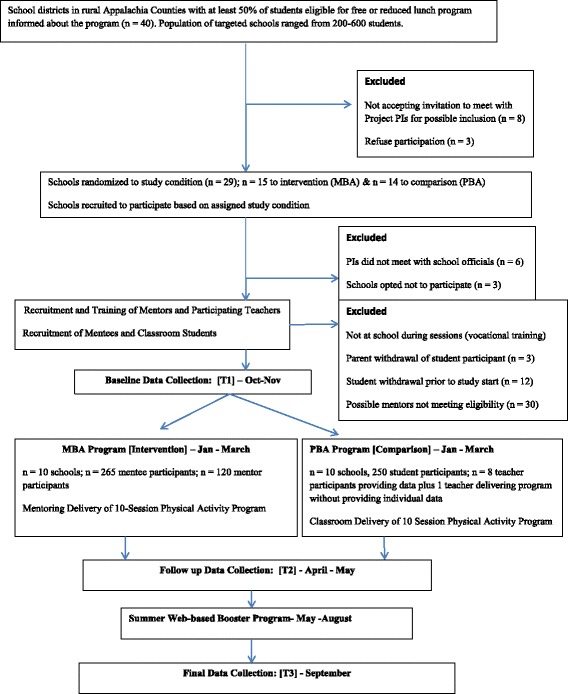
Experimental Design Overview

As a general measure of socioeconomic status, more than half of the adolescents attending these schools qualify for free or reduced lunch programs. Recruitment of mentor and mentee participants will occur during the start of the school year (September). With an average of over 60% mentee participation rate of eligible students (based on our preliminary work), we estimate that a total of 600 9th and 10th grade participants will be recruited over 3 years. Further, at least 100 older teens will be recruited to serve as peer mentors (10 per school × 10 schools) in the MBA condition, for a study total of 700 high school-aged children. See Fig. 2 for recruitment and design overview. To date, 119 older peer mentors and 654 9th graders have been recruited, exceeding our estimates. Seven classroom teachers have participated to date; four additional classroom teachers have been recruited to participate in Wave 3.

### Eligibility criteria for peer mentors and classroom teachers

Peer mentors will be recruited based on interest in working with peers, supporting others, and striving to cultivate their own health-supportive behaviors. Eleventh and 12th graders attending the target high school at the study’s start will be eligible to participate as mentors if they are not expected to move from the participating school before the conclusion of the study; can speak English; and are recommended by a teacher, school advisor, or counselor. Teens interested in serving as mentors will complete an application form; selection will be based on: study needs, motivation to serve, and recommendations. Potential teen mentors with a BMI (for age and gender) above the 85th percentile or below the 5th percentile at the start of the study will be excluded because of concerns about those not classified as having a healthy weight serving as role models for healthy lifestyle behaviors. These exclusionary criteria will be clear in recruitment materials so those excluded understand why. To date, thirty mentor applicants have been excluded because of not meeting eligibility criteria.

All teen mentors will attend the same school and reside in the same local community as study participants. Each peer mentor will be assigned up to 4 adolescents (2 per session; 2 possible sessions each week); based on school size and the number of mentees we will recruit 10–15 peer mentors per school. We will attempt to match mentors and mentees by gender.

School health and physical education teachers at participating schools will provide the comparison delivery method. With approximately 25 students per classroom, we anticipate 2 classrooms to participate in each comparison school. Based on preliminary studies, an 80% retention rate is expected, resulting in 82.5% power to detect a modest intervention effect. To aid retention of subjects, monetary incentives at each data collection time-point and booster kits for use during the summer months will be provided. In addition, attendance will be taken at all sessions; the PD will contact all subjects missing a session within 48 h. Reminders will be sent home to subjects.

### Intervention procedures

Intervention procedures include mentor training, curricular training, session or lesson protocols, incentive payments, and fidelity assessments. Steps to maintain curricular integrity are discussed.

#### Training

To maintain curricular integrity, each peer mentor and participating teacher will be trained in curriculum delivery and provided with an Instructor’s Guide that contains: (a) program overview and purpose, (b) weekly lesson plans, (c) cues and prompts to deliver content, (d) structured activities, (e) student workbooks containing all assignments, (f) content summaries to end each session, (g) copies of forms and handouts, (h) contact information for study personnel, (i) session locations, and (j) research protocols. The use of the Instructor’s Guide serves as a reference/reminder to the teen mentors and teachers of the message(s) to be delivered and support to be provided to participants. Curricular consistency is ensured through the structured training of the mentors and teachers that will include training courses for PBA or MBA lasting ~ 4 h for the curriculum they are to deliver.

In recognition of participant burden and consistent with NIH policy on participant compensation, peer mentors can receive up to a $330 incentive distributed as follows: $20 at completion of mentors’ training, $15 at completion of each intervention session (maximum two per week), and $10 for the return of supplies and materials. Curriculum and mentoring training will be conducted over a 4-h period. The PD will attend all training sessions. Peer mentor training follows the *Developmental Mentoring Training Methods* developed by Michael Karcher at University of Texas-San Antonio but adapted for use (with permission) for this project to focus on lifestyle patterns specific to Appalachia and mentor-mentee triads [49]. Training stresses the provision of tailored support using both didactic and experiential methods, such as role-play and demonstration. Components of mentor training include: mentoring responsibilities, sharing and working with different points of view, role-playing, and motivating mentees. Reinforcement of employing autonomy-supportive methods and messages will be continually stressed during the training. Also, peer mentors will be trained on curricular delivery of MBA. At weekly debriefing sessions all teen mentors will be asked to discuss experiences and provided a chance to ask questions and problem-solve concerns or issues.

All participating teachers in the PBA group will be local to the community and assigned to teach health and/or physical education to high school-aged students. Each teacher will deliver the PBA during a classroom session per week during health or physical education courses. Each teacher may lead 2 different sessions per week. Additional study-related training for PBA and its incorporation into the courses will be conducted over a 4-h period at each PBA school. The PD will attend all training sessions. During the curricular training session feedback and manualized materials will be provided. Although not all students enrolled in the health or physical education course may volunteer to participate in the study, the teacher will be provided curricular materials for all students in their courses.

#### Curricular protocol

Curricular workbooks will be provided to each participant containing the curriculum, manipulation checks (homework), worksheets, reinforcement activities, and weekly goal setting. Each week, participants consider ways to incorporate physical activity into their discretionary time. Care is taken to tailor the activity to each person’s interests, talents, and environment. The approach empowers adolescents to plan, regulate, and evaluate their personal activity plan. PBA has been refined over 4 pilot studies with Appalachian youth [17–19, 23]. These studies revealed PBA’s effectiveness at producing consistent changes in targeted SCT theoretical constructs and increase in moderate-intensity activity among previously sedentary students. PBA will be integrated into personalized mentoring (MBA) so adolescents will receive detailed, tailored instruction on the integration of physical activity into daily lifestyles as well as enhanced social support for behavioral change.

Mentors/mentees will be matched according to gender. Each participating high school will host the program on a different day of the week. The 11th and 12th grade teens will be assigned 9th or 10th grade mentees to meet within a large room (such as a gymnasium) where other triads (mentor and two mentees) are present. To minimize the number of trained mentors needed, each school assigned to the mentoring delivery will host a separate group on two different days of the week. Each mentor may engage in program delivery two days per week. The intervention group will receive the MBA curriculum via peer-to-peer mentoring during 40-min sessions.

To minimize distractions, mentor-mentee triads will not join any other triads (e.g., forming groups of 6) during curricular delivery. This workload was tested and found acceptable in preliminary studies. The mentoring triads will be distanced from other triads as much as feasible during curricular delivery. The PD will monitor the room for excessive loudness and instruct mentors to keep conversational voices if needed. Other outside distractions will be monitored by the PD and controlled as much as possible via limiting access to the room during the sessions. During the mentoring session, a PD will monitor all mentor/mentee interactions. All mentors will be provided a structured Discussion Guide to for each session. All triads will remain fully visible to the PD. It is projected that with 20 participating high schools, approximately 19 mentors will meet with two assigned students during each after-school session.

The comparison group with PBA lessons delivered via classroom teachers controls for the time spent with participants and content of the intervention. This design allows us to determine the efficacy of the mentored delivery (MBA) for this population. The 10-week PBA program will assist in ruling out alternative explanations of the delivery mechanism by which the intervention works. It will be standardized like the intervention curriculum, but delivery will differ.

At the comparison schools, PBA will be delivered as originally designed and tested: by classroom teachers during 20-min interactive sessions in existing health or physical education classes. All students enrolled in the participating courses will receive the PBA curriculum. Data will not be collected on any students enrolled in the health or physical education courses receiving the PBA curriculum who do not assent to participate in this study. Each teacher may lead 2 different classroom sessions per week during health or physical education courses. Based on 3 preliminary studies, we expect a high teacher retention rate (100% on all prior studies). If a teacher leaves the study before completing the intervention, a new teacher will be recruited and trained to deliver the remaining content. The possible replacement teacher will be identified after consultation with the school principal. If the replacement teacher is unable to deliver the remaining content during the students’ regular class time, another time during the school day, such as study hall period, will be used.

#### Fidelity assessment

To support curricular fidelity, mentors and teachers will collect homework from participants weekly. Homework will be reviewed by project staff for completeness and application of concepts and skills. Study participants will begin each session by writing the number of days they reached their physical activity goal(s) on the Project Poster Calendar.

To ensure program and study integrity, teen mentors and teachers will meet weekly (in separate sessions) with the PD for debriefing. During these 15-min debriefing sessions, the PD will: assess message consistency; reinforce follow-up messages; provide prompts and review content for the next week; and troubleshoot concerns. Curricular non-compliance and redirection will be discussed during weekly debriefing sessions. A PI will conduct curricular re-training to a mentor or PBA teacher who has more than one instance of curricular non-compliance. The PD will supervise the weekly teen mentor/mentee interactions and teacher/participant interactions, completing a *Measures of Fidelity* form for each observation.

The PD may terminate any interactions due to any participant objections. Should a mentoring interaction terminate, the PD will immediately contact a PI. With parental permission, the mentee and mentor will be interviewed separately about the terminated interaction. A parent may be present during the interview. Based on the interview’s outcome, the PI may reassign the participant to another mentor. The PD may be present at all future interactions between the deliverer of content and the participant; the data for this mentor/mentee dyad will be excluded from analysis. A PI will meet biweekly with the PD to review (a) curricular consistency among deliverers and (b) compliance with procedures and protocol. To further assess program fidelity, 25% of sessions will be randomly selected for video-taping and analyzed. Finally, a PI will “drop in” to observe both mentor-led and teacher-led sessions at least monthly, completing a *Measures of Fidelity* form.

### Measures

Measures are composed of items adapted from published studies and preliminary studies. All measures have been used with adolescents residing in rural or Appalachian settings and show acceptable psychometrics: internal consistency reliability α of .75–.94; face validity, predictive validity, and content validity, and/or construct validity are established. Participants and peer mentors will complete a demographic questionnaire with: age, birthdate, grade in school, gender, race and ethnicity, zip code, and household members.

### Primary health outcomes

Using the Tanita DC-430 U Body Composition Analyzer [50], Body Mass Index will be calculated for age and gender. The Tanita portable professional grade BIA analyzer has been found to be valid and reliable in estimating body mass index and the percentage of body fat in adolescents when compared to dual-energy x-ray [51, 52]. Height will be obtained by having the adolescent stand without shoes on a portable stadiometer facing forward. Individual’s age, gender and height are entered into the body composition analyzer for calculations. Participants stand on the body composition analyzer without shoes or socks, having their feet on the measuring pads and hands directed down the side of their legs.

#### Body fat percentage

Using body fat ranges for standard children [51–53] body fat percentage will be measured as the amount of body fat as a proportion of body weight. Proportion for age and gender was calculated by the Tanita DC-430 Body Composition Analyzer. Body fat percentage is estimated via the DXA method using the Bioelectrical Impedance Analysis Method [50–53]. Standard measurement modes were selected to obtain the most reliable results [50–53].

#### Body mass index percentile for age and gender

Using the CDC Teen Calculator, each participant’s date of birth, gender, day of data collection, weight to the nearest 1/8th pound, and height in feet and inches to the nearest 1/8th inch will be entered. Using sex-specific CDC guidelines for age, “underweight” is defined as below the 5th percentile; “healthy weight” is between the 25th and 85th percentile; “overweight” between the 85th and 95th percentile; “obese” is above the 95th percentile; and “extreme obese” is at or above the 120th percentile [54–58].

### Behavioral outcomes

#### Daily physical activity

Student, peer mentor, and teacher participants will wear accelerometers for 7 straight days (1 week) of physical activity data collection for each data collection cycle. The data will be used to estimate time spent in sedentary, moderate, and vigorous activity. Readings at or above 3962 counts per minute will be treated as vigorous physical activity [59–62]. Moderate physical activity cut points are 1535–3961 counts per minute [62]. Readings 100–1534 counts per minute are light activity [62]. Readings less than 100 counts per minute will be treated as sedentary activity [59, 61–63]. Two or more hours of zero counts suggests that device was not worn and will be excluded from sedentary analysis [59, 63].

### Psychosocial determinants

#### Outcome expectations for physical activity

The outcome expectancy values instrument assesses outcome expectations and their associated expectancies for physical exercise by requesting information on eight dimensions: relaxation, fitness, competition, social growth, social continuation, thrills, expressive movement, and beautiful movement. Each of the eight dimensions is measured by five items. Previous studies demonstrated internal consistency, reliability coefficient ranging α = 0.86–0.97 when used with Appalachian teens [64, 65]. Construct validity has been demonstrated through three confirmatory factor analyses using data from our previous work [61, 62].

#### Self-efficacy for physical activity

Self-efficacy will be measured using a previously developed instrument with 8 items [66]. The instrument has demonstrated predictive validity for boys and girls: 0.23 and 0.27 [66]. Re-test reliability of this scale has been reported to be 0.82 [61, 62]. This instrument has been refined by adding three additional items and altering the response scale from dichotomous to a five-point Likert-type-type scale, with internal reliability consistency ranging α = .85–.94 [64, 65].

#### Self-regulation for physical activity

This measure contains six subscales: Behavioral Goal Setting, Self-Monitoring, Overcoming Barriers, Time Management, Self-Reward, and Planned Social Support [67]. The instrument was developed by Petosa using a three-stage expert panel review to establish content validity. Internal reliability ranged from 0.89 to 0.95 [64]. Construct validity has been demonstrated through confirmatory factor analyses [67].

#### Social support

Social support is measured using a self-report questionnaire containing eight items originally developed by Reynolds et al. [68] and refined by Trost et al. [69]. This instrument measures instrumental social support, social encouragement, and social expectations that are provided by friends and family members for physical exercise. To increase the internal reliability of the instrument, the original reporting scale was expanded to a five-point Likert-type scale [64, 65]. This instrument has previously been demonstrated to have construct validity [67, 68]. Internal reliability ranged α = .75–.88, and re-test reliability ranged *R* = 0.78–0.93 [61, 62].

### Environmental determinants

#### Perceived environment for physical activity

Perceived environment is measured by two Likert-type sub-scales. The first 9-item subscale focuses on the perceived home environment. The second 10-item sub-scale focuses on the perceived neighborhood environment. Construct validity and test-retest reliability have been established for both scales when used with adolescents residing in rural communities [69].

### Data procedures

Prior to baseline data collection, a series of training sessions will be held to train data collection staff responsible for taking measurements, including physical activity using Actigraph wGT2X-BT accelerometers [70], BMI, body fat, and administration of the data collection instruments. The measurement team training will be held at the Project Office. To assure standardization and quality of data collection, this training will include a review of the eligibility criteria and consent procedures; overview of the measurement protocol; demonstration of the measurement methods; and an opportunity to have each measurement team mock data collect on several subjects and gain expert feedback on their ability to follow protocol. This training will occur prior to each data collection time period (T_1_, T_2_, and T_3_).

The Project Office will ensure that the measurement teams perform only those functions for which they are certified, and that re-certification activities are implemented as planned in a timely manner. The host institution maintains an Appalachian Translational Research Office in the local region, employing local residents. Staff from the center will be hired as research assistants (RAs, master’s-prepared residents trained in data collection procedures and blinded to study purposes, group assignment, and treatment or control groups) to conduct data collection for this project. RAs will collect anthropometric and other quantitative data in a designated private room at each participating high school at all data-collection time-points (T1, T2, and T3). Anthropometric data will be collected individually in a private room separate from the survey room, such as the school clinic. Pilot testing found anthropometric data collection will take 10–15 min.

Written surveys will be administered in a group setting at the beginning of the designated class period using a paper-and-pencil format. Students will be provided their own desk space. Filling out the complete battery of surveys takes approximately 20 min. Each RA will read explicit directions regarding how each survey will be filled out. With each instrument, the RA will read the directions, answer questions, and then allow students to fill out the instrument. Each RA will available to answer questions. When the entire battery is completed, the measurement team will collect the data, count the surveys, and record the count on a data sheet that is placed in a box and sealed.

Collection of the physical activity variable requires that the Actigraph be worn by the study participants for seven study days, and requires two school visits by the measurement team for each participant. There will be a demonstration of the correct right iliac crest placement of the monitor using a belt provided by the study. Each subject will receive a belt that allows for the correct placement of the monitor and written directions for the subject and parent to assure correct placement of the device in subsequent days. The participants will receive a magnet to take home as a reminder on proper use and wearing of the device during the monitoring period. After seven days of monitoring, the measurement team will return to the school to collect the devices, debrief the participant, and provide an incentive.

#### Data storage

Demographic information or identifiable data will be removed immediately from the surveys, transported to the project office, and stored separately from the surveys. Once completed, all written surveys and paper data forms will be placed in a sealed plastic container secured in the locked trunk of the car for transport back to the Project Office. The sealed container will not be opened until it reaches the Project Office. Once opened, the surveys will be counted to verify there are no missing forms. Accelerometer data will be downloaded to a portable laptop and data saved on a password-protected and encrypted external hard drive for transport to the research office. No data will be saved on the laptop or other portable devices. Data will be saved to the server at each data entry time-point. Per policy, the server is backed up at least every 24 h. Interview notes, audio-taped recordings, and transcription notes will be stored in a locked cabinet in the Project Office. Access to data will be limited to IRB-approved project staff.

#### Data management

Teen participants (mentors, students, and teachers) will receive $15 at each data-collection time-point. Anthropometric measures (height, weight, and body composition) will be collected from each privately. Data from peer mentors and teachers will be collected by an RA at completion of curricula training (T_1_), at the end of the academic year (T_2_), and beginning of the following academic year (T_3_). Informed consent will be documented on a tracking form that allows linkage of the participant to their study identification number. For subsequent data collection, the identification number will be used instead of any identifiable information. All databases will be encrypted and password-protected. We will use a randomly generated registration and tracking number to document all measurement activities within individuals. Once informed consent is given, information on physical activity levels will be collected using the Actigraph accelerometer.

### Statistical models and methods of analysis

#### Approach

A positive intra-class correlation (ICC) is expected among students in the same school due to commonalities in selection, exposure, mutual interaction, or a combination of those factors. Ignoring positive ICCs can inflate Type 1 error rate in a G-RCT [71–76]. These problems will be avoided by analytic methods appropriate to the structure of the design and data. Specifically, we will fit Linear Mixed Models (LMMs) and Generalized Linear Mixed Models (GLMMs) to account for various levels of correlation among participants [77]. We will fit these models using Stata.

#### Aim 1

Aim 1 outcome variables measure BMI and body fat. For each of these measures, we will fit a mixed-model ANCOVA to account for the correlation of students within schools and improve statistical efficiency by adjusting for the individual baseline value of the outcome. The fixed effect of group will be the average difference in BMI (or body fat) at T_3_ between the mentor-led (MBA) and teacher-led (PBA) groups.

A sensitivity analysis will compare the results at T_2_ between the groups, again adjusting for baseline values in a mixed-model ANCOVA. We will assess dose-response within each group by using an interaction model. In this mixed model, the outcome variable is BMI and the primary predictors of interest are group (MBA or PBA), the number of sessions attended (in either group), and the interaction between the number of sessions and group. This model will also adjust for baseline BMI and include a random effect of school to account for the correlation of outcomes from students in the same school. In this model, we expect the coefficient for the interaction term to be significant, indicating a significant difference in the effect of number of sessions attended between the two groups. We expect the estimated decrease in BMI for each session attended to be larger in the MBA group, indicating a stronger effect with fewer sessions.

#### Aim 2

The analysis of Aim 2 is similar to that of Aim 1, except the outcome data are physical activity (daily physical activity, exercise, sedentary activity). These outcomes are also continuous, so the statistical approach is identical to Aim 1. Using accelerometer data, we will compare the average daily total physical activity and the average time spent in exercise, defined as moderate/vigorous physical activity [78] (MVPA) and sedentary activity (< 100 counts per minute) per day between two groups. To estimate the main effect of the intervention, we will again use a mixed-model ANCOVA, in which models are adjusted for the baseline value of the outcome. The fixed effect of group will be the average difference in physical activity (or MVPA) at T_3_ between the mentor-led (MBA) and teacher-led (PBA) groups. We will similarly assess dose-response within each group using a mixed-model ANCOVA stratified by group. In these models, we expect the coefficient for the number of sessions attended will be positive (indicating greater physical activity among those students who attended more sessions) and that the coefficient for number of sessions will be even more positive among the MBA group, indicating greater effectiveness with fewer sessions. Models will be adjusted for baseline physical activity values and for the correlations of students/same school by including a school random effect.

#### Aim 3

In Aim 3, the goal is to estimate the amount of change in physical activity between baseline (T_1_) and the end of the study (T_3_) among the peer mentors. In this analysis, there is no comparison group, so the outcome variable is the difference in physical activity (or MVPA), and models will adjust for a random effect of school to account for similarities between mentors from the same school.

#### Assumptions

The mixed models assume that there are two sources of random variation: schools and individuals. The observations are assumed independently conditional upon these random effects and values of the covariates. There are additional assumptions inherent in regression (e.g., linearity of effects, homogeneity of variance); we will check those assumptions.

#### Intention-to-treat

The primary analysis will follow intention-to-treat principles [73, 79–81]. Randomization carries the expectation that the study conditions will be equivalent at pretest with respect to known and unknown prognostic factors. As a result, removing randomized groups or members from the analysis runs the risk of tampering with this balance and introducing bias. Further, loss of one or more groups could create an unbalanced design at the group level and heighten the risk associated with heteroscedasticity in a G-RCT [73]. Based on our previous research, we estimate that no more than 20% of members measured at pretest will be missing at posttest, although we will make every effort to obtain posttest data on all individuals, including those who stop attending the sessions. Multiple imputations are now widely regarded as an effective method for replacing missing data [82, 83] and we will use this approach, adapted for use with a G-RCT [84–86].

#### Multiple comparisons

The primary analysis is the difference in BMI between the two groups and in the power calculations. Our analysis of the accelerometer data will focus on changes in the daily physical activity at T_3_. For each aim, we have one primary comparison, and each will be conducted at the two-sided 0.05 level. All other analyses will be secondary and will be reported as such; therefore, no adjustments will be made for multiple comparisons.

## Discussion

The long-term goal of this study is to positively impact the physical activity patterns to improve health outcomes including the high rates of obesity in Appalachian teens. Our innovative approach will train peer mentors to deliver the culturally appropriate intervention and provide social support that is critical for facilitating and sustaining health behavior change. This study is innovative by: (a) providing age-appropriate lifestyle education and skill building (goal setting, engaging in regular physical activity and exercise, self-regulation, and building self-efficacy); (b) providing peer-to-peer mentoring by local high school students and school-based tailored support to change behaviors; (c) focusing on unique healthy-lifestyle challenges (lack of organized sports and recreational facilities) prevalent in low-resource areas such as Appalachia; (d) overcoming environmental, social, and psychological barriers to improve adherence to physical activity; and (e) increasing teen resources and support (social network and relationships) to perform the targeted behaviors. We predict that by serving as role models, peer mentors will improve their own lifestyle behaviors, providing a duel intervention [33, 34, 45]. We predict that providing individual and structured social support to teens via peer mentors will result in better health outcomes compared to teacher-based support alone (usual care).

This study will advance scientific knowledge and clinical practice in several ways**.** First, the proposed study will evaluate the efficacy of PBA delivered via peer-to-peer mentoring by trained high school teens on short- and long-term outcomes in an understudied, underserved, under-resourced, and low-income population. Second, by using local trained peer mentors to serve as role models and deliver the intervention program, our study provides a double-pronged intervention affecting the health behaviors of mentors and expanding the reach in the community. Third, our aims will contribute to understanding the appropriate intervention dose needed to achieve meaningful changes in health behaviors and health outcomes. Our aims ultimately will guide the development of effective interventions specifically targeting residents of Appalachia, a region with disproportionally high prevalence rates of childhood obesity, low physical activity levels, and challenges to achieving and sustaining healthy lifestyles.

This project seeks to shift intervention research and practice away from a disease treatment model and toward a health promotion and prevention framework focused on promoting healthy behaviors in a population-based sample of adolescents. In under-resourced schools striving to meet academic mandates, school administrators seek flexible alternatives to classroom-based health and physical education. If our hypotheses are supported, the project will serve as a model for working with under-resourced and other unique or under-served adolescent populations.

Taking the results from this trial, we have the possibility of developing a wider scale trial targeting broader rural, underserved areas or hard to reach populations. The intervention may be even more beneficial to younger Appalachian adolescent populations such as middle-school age students being mentored by high school peer mentors. Broad dissemination of an efficacious, community based and community-driven intervention has the potential to mitigate adolescent obesity rates and its co-morbidities among this population.
